# Strukturierte Cloud Transformation in Unternehmen – Veränderungen durch Covid-19?

**DOI:** 10.1365/s40702-021-00742-y

**Published:** 2021-06-24

**Authors:** Fabian Brechlin

**Affiliations:** 1 IT-Beratung & Services – Rewion GmbH, Fichtenweg 6, 71711 Murr, Deutschland; 2 Duale Hochschule Baden-Württemberg DHBW, Stuttgart, Deutschland

**Keywords:** Cloud Computing, Digitale Transformation, Cloud-Technologie, Cloud Transformation Roadmap, Change Management, Cloud computing, Digital transformation, Cloud technology, Cloud transformation roadmap, Change management

## Abstract

Die Verlagerung von On-Premise Infrastruktur und Workflows in die Cloud ist schon lange kein Trend mehr, sondern eine gängige und notwendige Unternehmenspraxis. Die Covid-19 Pandemie hat vielen Unternehmen gezeigt, was für eine zentrale Rolle die Nutzung von Cloud-Services heutzutage spielt. Auch wenn sich die Cloud Transformation an sich durch die Pandemie nicht grundlegend ändert, so wird sie doch vielerorts erheblich beschleunigt oder, falls noch nicht begonnen, angestoßen. Ortsunabhängiges Arbeiten, flexible IT-Strukturen und digitale Kollaborationsmöglichkeiten sind heute oft notwendig, um einen erfolgreichen Betrieb zu gewährleisten. Die Nutzung von Cloud-Services ist dadurch fast unumgänglich geworden.

Dieser Artikel basiert auf zahlreichen Praxiserfahrungen und Beobachtungen aus Cloud-Projekten bei unterschiedlichen Unternehmen. Er zeigt auf, welche Aspekte und Herausforderungen bei einer ganzheitlichen und gut geplanten Cloud Transformation berücksichtigt werden sollten. Dabei wird Bezug auf das Coronavirus als zusätzlicher Cloud-Treiber genommen. Die „Cloud Transformation Roadmap“ als Leitfaden für die Etablierung der Cloud wird vorgestellt, ebenfalls wird auf die Wichtigkeit eines ganzheitlichen Change Managements eingegangen. Es werden konkrete Empfehlungen für eine erfolgreiche Meisterung des Cloud-Einstiegs gegeben.

## Abstract (en)

Moving on-premise infrastructure and workflows to the cloud is no longer a trend, but a common and necessary enterprise practice. The Covid-19 pandemic has shown many companies what a central role the use of cloud services plays today. Even if the cloud transformation itself does not change fundamentally because of the pandemic, it will be accelerated considerably in many places or, if not already started, triggered. Location-independent working, flexible IT structures and digital collaboration options are often necessary today to ensure successful operations. As a result, the use of cloud services has become almost inevitable.

This article is based on numerous practical experiences and observations from cloud projects at different companies. It shows which aspects and challenges should be considered in a holistic and well-planned cloud transformation. Reference is made to the corona virus as an additional cloud driver. The “Cloud Transformation Roadmap” as a guideline for establishing the cloud is presented, the importance of holistic change management is addressed as well. Concrete recommendations for successfully mastering cloud entry are given.

## Die Covid-19 Pandemie: Augenöffner oder Beschleuniger für die Cloud?

Seit über einem Jahr hat die Covid-19 Pandemie die Welt fest im Griff. Die zahlreichen Maßnahmen zur Eindämmung der Virusausbreitung haben auch für die Arbeitswelt massive Veränderungen mit sich gebracht. Wo immer es geht, arbeiten die Menschen im Homeoffice, Geschäftsreisen fallen größtenteils weg, Messen finden online statt – die Bedeutung der Digitalisierung hat noch einmal kräftig zugenommen. Für Unternehmen gilt es, ihre internen Prozesse durch geeignete Workflows so umzustellen, dass sie Homeoffice-tauglich sind. Im ersten Schritt bedeutet dies, Prozesse zu digitalisieren, im zweiten Schritt kann dann eine Cloud-Lösung zum Einsatz kommen.

Unternehmen, die bereits vor der Pandemie auf den Zug der Digitalen Transformation aufgesprungen sind, hatten in den letzten Monaten einen klaren Vorteil. Die Verlagerung von Workflows in die Cloud ist nichts Neues – viele Unternehmen haben bereits vor Beginn der Pandemie Cloud-Lösungen etabliert oder mit deren Einführung begonnen. Die technischen Voraussetzungen für die neuen Anforderungen an Arbeitsweise und -umgebung waren hier bis zu einem gewissen Grad bereits gegeben. Eine entsprechende Anpassung konnte relativ schnell und flexibel umgesetzt werden. War die Cloud Transformation noch nicht so weit vorangeschritten, dann wurde diese durch die Pandemie erheblich beschleunigt.

Für die Unternehmen, welche sich bisher noch nicht aktiv auf den Weg in die Cloud begeben haben, war und ist Covid-19 ein Augenöffner. Das Bewusstsein für die Notwendigkeit, möglichst umfassend digital und ortsunabhängig interagieren zu können, wurde spätestens durch die Pandemie geweckt. Unternehmen werden nun gezwungen, sich zu überlegen, inwiefern Infrastruktur und Prozesse in die Cloud verlagert werden können. In der Praxis zeigt sich das oft so, dass die Initiative für den Cloud-Einstieg inzwischen von der obersten Geschäftsleitung kommt. Wurde der CIO vor ein paar Jahren noch gebremst, wenn es um die Cloud ging, so wird er heute explizit vom CEO gefragt, wie weit das Unternehmen bereits mit der Cloud ist.

Das Thema Cloud ist durch die Pandemie nicht neu entstanden – die Relevanz der Cloud ist jedoch noch einmal deutlich gestiegen. Das zeigen auch neueste Studien, z. B. der „State of the Cloud“ Report 2021 von Flexera. Demnach setzen 89 % der befragten 155 europäische Unternehmen durch die Covid-19 Pandemie stärker auf die Cloud als vorher (Flexera 2021, S. 68). Unter den 750 weltweit befragten Unternehmen gaben 90 % an, dass sie die Cloud aufgrund der Pandemie intensiver als gedacht nutzen (Flexera 2021, S. 10).

Auch die Vorteile, die eine cloudbasierte Umgebung mit sich bringt, wurden klar ersichtlich. Eine Cloud Transformation bringt jedoch auch zahlreiche Herausforderungen mit sich und benötigt eine ganzheitliche und gut organisierte Planung. Dies sollten Unternehmen auch bei einer beschleunigten Einführung der Cloud nicht außer Acht lassen, um zukünftig eine effiziente und zielführende Nutzung der Cloud zu gewährleisten.

### Was verstehen Unternehmen unter „Cloud“?

Wenn heute von der Cloud gesprochen wird, ist es wichtig, von Anfang an abzuklären, was die Beteiligten unter diesem Begriff verstehen. Abhängig von individuellen Erfahrungen und Berührungspunkten, kann „Cloud“ eine andere Bedeutung haben. Wird ein bestimmter Anbieter oder eine bestimmte Lösung darunter verstanden? Auf welches Service-Modell wird Bezug genommen? Diese Fragen gilt es zu Beginn eines Cloud-Projekts zwingend zu klären.

**Abb. 1:**
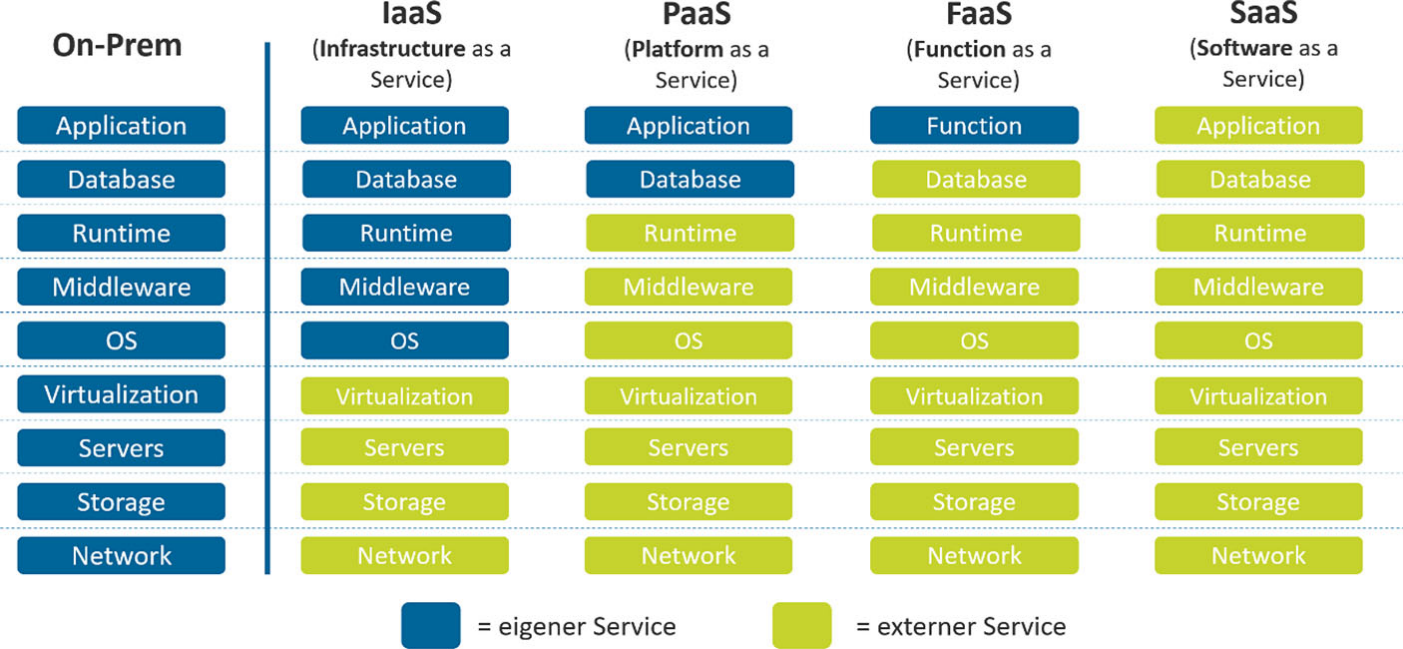
Übersicht über die unterschiedlichen Cloud-Service-Modelle (eigene Darstellung)

Cloud-Services werden üblicherweise in vier unterschiedliche Ebenen bzw. Service-Modelle unterteilt (siehe Abb. 1). Je nach Cloud-Service-Modell werden mehr oder weniger Zuständigkeiten an den Cloud Provider übertragen. Während bei IaaS (Infrastructure as a Service) lediglich die Hardware-Ressourcen durch Cloud-Services abgedeckt werden, umfasst SaaS (Software as a Service) zusätzlich die cloudbasierte Software-Nutzung. Auch die komplette Wartung und die Hintergrund-Administration werden bei SaaS vom jeweiligen Anbieter übernommen. Beim PaaS Modell (Platform as a Service) wird eine Entwicklungs- und Testumgebung bereitgestellt, um eigene cloudbasierte Services zu entwickeln und bereitzustellen. FaaS (Function as a Service) ist dem „Serverless Computing“ einzuordnen. Hier wird ausschließlich die Geschäftslogik selbst verwaltet. Ein bekanntes Beispiel sind die Skills von Amazons Sprachassistentin Alexa (Gill 2015, S. 17–18).

Diese Unterteilung verdeutlicht: Cloud ist nicht gleich Cloud. Je nach Struktur, Zielen und Grundsätzen kann sich ein anderes Service-Modell besser eignen. Durch das Verständnis für die unterschiedlichen Modelle können verschiedene Meinungen und Vorurteile zum Thema Cloud besser verstanden und abgegrenzt werden.

### Die Pandemie als zusätzlicher Cloud-Treiber

Für den Einstieg in die Cloud gibt es viele Gründe. Aus Sicht der Cloud-Anbieter sind dies das Auslaufen von Daten-Center Verträgen, die schnelle Integration von Akquisitionen, der dringende Bedarf an zusätzlichen Kapazitäten, die Erneuerung von Soft- und Hardware, eine verbesserte IT-Stabilität, Compliance-Gründe, Anwendungsinnovationen, das Ende eines Software-Supports oder die Verbesserung der CO^2^-Bilanz (Microsoft 2019).

Die Praxis zeigt: Bei den Unternehmen gibt es vier Hauptgründe, welche die Cloud Transformation vorantreiben:

1. **Flexibilität:** Die Cloud ermöglicht eine flexible Anpassung von IT-Strukturen, sodass sowohl Prozesse, aber auch Geschäftsmodelle, schnell an neue Gegebenheiten angepasst werden können. Ebenfalls können IT-Ressourcen flexibel eingesetzt werden.
2. **Innovation:** Unternehmen nutzen Cloud-Lösungen als neue Technologie, um Innovationen voranzutreiben. Viele technische Innovationen gibt es heutzutage nur als Cloud-Service, z. B. Sprachassistent*innen (Alexa von Amazon, Siri von Apple etc.) oder Anwendungen in den Bereichen künstliche Intelligenz und maschinelles Learning (z. B. Google Cloud-Anwendungen) – wollen diese genutzt werden, ist die Arbeit mit der Cloud eine zwingende Folge.
3. **Bereitstellungszeit:** Durch die Nutzung von Cloud-Diensten sind Unternehmen dazu in der Lage, neue Service-Angebote auch kurzfristig zu realisieren. Hier kann zum Beispiel der „Greenfield“-Ansatz^1^ genutzt werden. Hier werden Prozesse unabhängig von bestehenden Strukturen komplett neu aufgebaut. Dadurch schaffen es Unternehmen, Cloud-Services innerhalb kürzester Zeit bereitzustellen.
4. **Kostenreduktion:** Der Wegfall von hohen einmaligen Ausgaben für die physische IT-Infrastruktur bietet einen zusätzlichen Anreiz für Unternehmen, in die Cloud zu wechseln.

Nun ist mit der Covid-19 Pandemie ein zusätzlicher Cloud-Treiber hinzugekommen. Die Aufrechterhaltung eines reibungslosen Betriebs ist bei Homeoffice-Regelungen oder Abwesenheiten aufgrund von Quarantäne mit reinen On-Premise-Lösungen nicht mehr möglich. Die durch Cloud-Lösungen mögliche digitale Zusammenarbeit, sowohl unternehmensintern als auch extern mit anderen Unternehmen, erleichtert den Arbeitsalltag in der aktuellen Zeit deutlich. Die ständige Verfügbarkeit von IT-Diensten ist für das effiziente ortsunabhängige Arbeiten und das Homeoffice unabdingbar.

## Die Cloud als Teil der Unternehmensstrategie

Die Cloud an sich ist keine Strategie. Vielmehr benötigt ein Unternehmen für die Cloud Transformation eine klare Strategie, ausgerichtet an der allgemeinen Unternehmensstrategie. Die Cloud aufgrund von Covid-19 nun voreilig bzw. ohne Plan einzuführen, sollte daher vermieden werden. Bei einem zu schnellen, unüberlegten Einsatz der Cloud kann diese höchstwahrscheinlich nicht effizient genutzt werden, was wiederum zu hohen Kosten führen kann.

Auch wenn die Cloud selbst nicht als Strategie bezeichnet werden sollte, kann ihr Einsatz die Folge eines (übergeordneten) strategischen Ziels sein, z. B. die Kostenumverteilung von Fixkosten zu variablen Kosten. Auch in diesem Fall wird für die Cloud Transformation eine eigene Strategie benötigt.

Ist eine Cloud-Strategie definiert und wird sie entsprechend verfolgt, kann die Cloud als umfangreicher Werkzeugkasten angesehen werden. Sie unterstützt Unternehmen bei der Potentialentfaltung und hilft ihnen dabei, mit neuen Geschäftsideen zu experimentieren.

### Der Start auf dem Weg in die Cloud

Wie kommt man in die Cloud? Einfach einen Account anlegen und los geht’s? Ganz so leicht ist es für Unternehmen nicht. Handelt es sich nicht gerade um ein Start-Up, das von Anfang an auf Cloud-Lösungen gesetzt hat, müssen meist viele Prozesse angepasst oder neu definiert werden. Bis eine effiziente Nutzung der Cloud möglich ist, benötigt es oft auch Zeit, um Erfahrungen zu sammeln und die entsprechenden Schlüsse daraus zu ziehen. Zudem sehen sich manche Unternehmen erst einmal einer Art „Cloud-Dschungel“ ausgesetzt. In der On-Premise-Welt waren Server, Netzwerkkomponenten, User etc. noch in jeweils eigenen Systemen verwaltet. In der Cloud befinden sich nun alle Komponenten an einer zentralen Stelle. Eine transparente Verwaltung an einem Ort ist zwar durchaus gewollt, bedarf aber dennoch eines guten Konzepts. Ansonsten besteht schnell die Gefahr, dass ein Durcheinander an Abhängigkeiten entsteht und es zu einem sogenannten „Cloud Sprawl“^2^ kommt.

Die Einführung der Cloud erfordert ein Umdenken bei allen betroffenen Menschen. Sie ist maßgeblich an einem Kulturwandel im Unternehmen beteiligt und betrifft alle Mitarbeitenden – nicht nur die IT-Abteilung. In vielen Unternehmen wurde dieser Kulturwandel durch die Corona-Pandemie zwangsweise angestoßen oder, falls er bereits im Gange war, beschleunigt. Hiervon kann die Cloud Transformation profitieren. Oft mussten Unternehmen und Mitarbeitende erleben, was es bedeutet, keinen digitalen Zugriff auf Prozesse oder Dokumente zu haben. Dies hat die Vorteile der Cloud-Technologie und die Notwendigkeit für deren Einsatz aufgezeigt.

### Herausforderungen bei der Cloud Transformation

Bei der Cloud Transformation geht es um das Unternehmen als Ganzes, nicht nur um die Einführung einer neuen Technologie. Neben der Technologie sind es vor allem die Menschen und die Prozesse, welche zentral für die erfolgreiche Cloud Transformation sind. Nur wenn diese drei Komponenten zusammenspielen und die komplette Organisation auf die Umstellung vorbereitet ist, kann die Cloud einen wahren Mehrwert bieten. Die Komplexität der Cloud Transformation bringt daher einige Herausforderungen mit sich.

- **Komplexität der IT-Landschaft:** Die IT-Landschaft eines Unternehmens wurde oft über Jahre hinweg aufgebaut. Entsprechend komplex ist nicht nur die technische Infrastruktur, sondern auch die gesamte Enterprise Architektur eines Unternehmens. Viele Unternehmen vollziehen den Wandel in die Cloud schrittweise und nutzen unterschiedliche Cloud-Anbieter (Multi Cloud). Zur unternehmenseigenen IT kommen externe Services hinzu. Hier gilt es, an einer zentralen Stelle die Übersicht zu behalten und Alleingänge von einzelnen Abteilungen zu unterbinden.
- **Umfassendes Prozess-Knowhow:** Alle Abhängigkeiten und Wechselwirkungen innerhalb der Unternehmensprozesse müssen bekannt sein, eine ausführliche Prozessanalyse ist daher unverzichtbar. Die Funktionalität wichtiger Schnittstellen zwischen den verschiedenen IT-Diensten muss auch nach der Einführung von Cloud-Lösungen gewährleistet sein. Hierfür sollte frühzeitig ein entsprechendes Konzept ausgearbeitet werden.
- **Klare Zieldefinition:** Die Anforderungen an die Cloud-Lösung(en) und die zu erreichenden Ziele müssen klar definiert werden und der allgemeinen Unternehmensstrategie entsprechen. Eine unklare oder gar fehlende Zielsetzung führt zu voreiligen Entscheidungen und damit zu unpassenden Cloud-Lösungen.
- **Change Management:** Die Umstellung auf die Cloud ist mit vielen Veränderungen verbunden und wirkt sich auch auf Ihre Unternehmenskultur aus. Neue Technologien werden genutzt, Prozesse werden angepasst und Mitarbeitende müssen alte Gewohnheiten ablegen, um die neuen Anwendungen zielgerichtet nutzen können. Fehlende Kompetenzen oder veraltete bzw. unpassende Unternehmensprozesse verhindern die erfolgreiche Nutzung der Cloud. Umso wichtiger ist es, rechtzeitig neue Kompetenzen aufzubauen, Mitarbeitende ausreichend zu schulen und die Organisation gründlich auf die Umstellung vorzubereiten. (Weitere Informationen zum Thema Change Management: siehe Abschn. 3.3)
- **Datensicherheit & Datenschutz:** Das Verhindern von Datenverlust sowie der Schutz von sensiblen Daten vor unberechtigten Zugriffen sind auch im Zuge der Cloud Transformation unabdingbar. Die meisten Cloud-Anbieter treffen hier umfassende Maßnahmen, teilweise können sie auch höhere Sicherheitsstandards gewährleisten als manche Unternehmen. Eine kritische Prüfung ist aber unbedingt notwendig. Zertifikate, Server-Standort des Anbieters und vorhandene Verschlüsselungsoptionen sollten zum Beispiel beachtet werden. Ebenso ist ein Bewusstsein über die Compliance-Anforderungen notwendig – zur Auswahl stehende Anbieter sollten diesbezüglich geprüft werden. Bei der Nutzung von Cloud-Diensten gilt die „Shared Responsibility“, d. h. nicht nur der Cloud Provider, auch der Cloud-Kunde trägt einen Teil der Verantwortung für die Sicherheit.
- **Performance Sicherstellung:** Durch die (teilweise) Migration der IT-Infrastruktur in die Cloud gibt ein Unternehmen auch ein gewisses Maß an Kontrolle ab. Ein Ausfall der Cloud-Dienste, verzögerte Antwortzeiten oder die eingeschränkte Cloud-Verfügbarkeit könnten im schlimmsten Fall sowohl Umsatz als auch Kunden kosten. Auch wenn Cloud-Lösungen genutzt werden, sollte weiterhin der Überblick über den Zustand und die Performance der eingesetzten Anwendungen behalten werden. Hier empfiehlt sich der Einsatz eines Cloud Monitorings. Dieses hilft bei der Überwachung der Workflows und Prozesse der Cloud-Dienste, sodass Probleme frühzeitig erkannt und behandelt werden können.

Falls ein Unternehmen international tätig ist, sollten vor dem Cloud-Einstieg außerdem immer die länderspezifischen Regelungen überprüft und in die Planung miteinbezogen werden. Hierbei sollten die gesamte Netzwerk-Infrastruktur sowie sogenannte lokale Internet-Breakout^3^ berücksichtigt werden.

Generell sollten sich Unternehmen bewusst sein, dass auch mit dem Einsatz der Cloud weiterhin viel Verantwortung bei ihnen selbst liegt. Je mehr Bereiche vom Cloud-Anbieter übernommen werden, desto mehr Verantwortung kann abgegeben werden. Bei einer SaaS Lösung liegen weniger Verantwortungen beim Unternehmen als bei einer IaaS Lösung (siehe Abschn. 1.1). Die Verantwortung für die professionelle Auswahl einer Compliance-konformen Cloud-Lösung sowie für eine reibungslose Zusammenarbeit mit dem Cloud-Anbieter trägt selbstverständlich immer das Unternehmen. Durch den Einsatz der Cloud verändert sich ebenfalls der Verantwortungsbereich von IT-Verantwortlichen, es werden neue Kompetenzen benötigt und teilweise auch ganz neue Stellen geschaffen (siehe Abschn. 3.4).

Mit der Corona-Pandemie ist die Zeit als eine zusätzliche oder größere Herausforderung der Cloud Transformation dazugekommen. Unternehmen werden durch Covid-19 unter Druck gesetzt, den Einstig in die Cloud möglichst schnell umzusetzen, um digitaler aufgestellt zu sein. Mit „digitaler aufgestellt“ sind hier ein ortsunabhängiger Betrieb, digitale Prozesse ohne Systembrüche sowie die effiziente und flexible Skalierung der Infrastruktur und Services gemeint. Gleichzeitig sollte die Cloud-Einführung aber gut geplant sein, um zukünftig eine effiziente und zielführende Nutzung der Cloud zu ermöglichen.

## Durchdachte Cloud Transformation mit Plan

Transformation bedeutet Wandel. Die Einführung der Cloud im Unternehmen bewirkt Veränderung bei eingesetzten Technologien, Prozessen und nicht zuletzt in der Arbeitsweise der Mitarbeitenden. Im Optimalfall bietet die Cloud einen individuellen „Werkzeugkasten“ (siehe Abschn. 2), der bei der richtigen Anwendung effizient im Geschäftsalltag und bei der Zielerreichung unterstützt. Die von der Rewion GmbH entwickelte „Cloud Transformation Roadmap“ bietet Unternehmen einen Leitfaden, der sie dabei unterstützt, aus den zahlreichen verfügbaren Möglichkeiten die richtigen Werkzeuge für die spezifischen Bedürfnisse zusammenzustellen.

### „Cloud Transformation Roadmap“ als Leitfaden

**Abb. 2:**
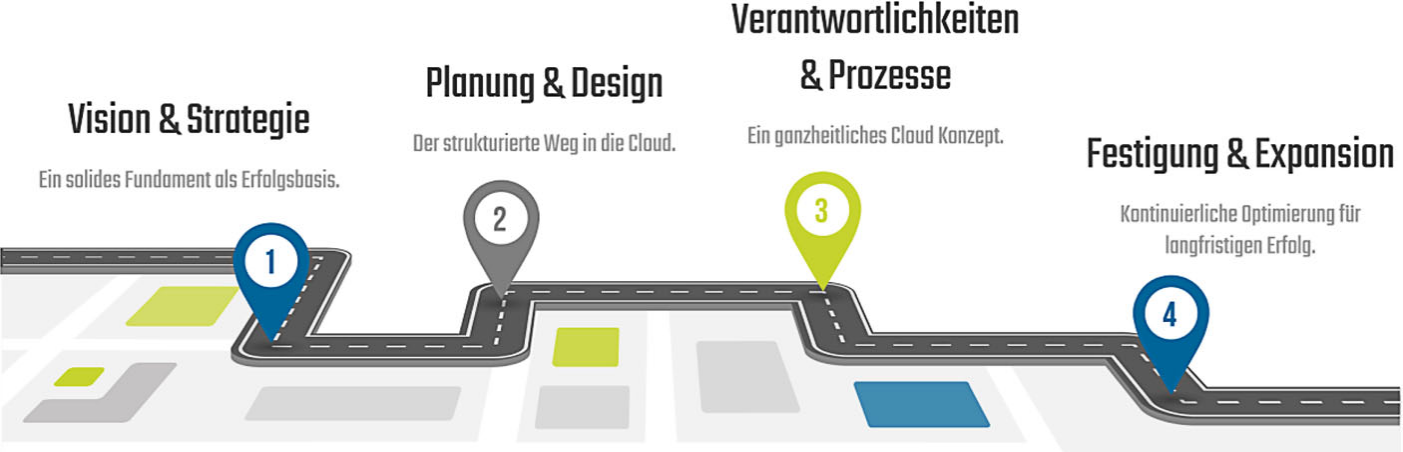
Die vier Phasen der „Cloud Transformation Roadmap“ (eigene Darstellung)

Die „Cloud Transformation Roadmap“ unterstützt Unternehmen dabei, ihren individuellen Weg in die Cloud zu finden und umzusetzen. Jedes Unternehmen hat andere Strukturen, Ziele und Grundsätze – daher muss auch der passende Weg in die Cloud unternehmensspezifisch evaluiert werden. Die „Cloud Transformation Roadmap“ besteht aus vier Phasen und gibt Unternehmen Orientierung, welche Themen beim Einstieg in die Cloud zu welchem Zeitpunkt beachtet werden müssen (Abb. 2).

In der ersten Phase der „Cloud Transformation Roadmap“ werden die Vision und die Strategie festgelegt, welche durch den Cloud-Einstieg verfolgt werden sollen. Hier wird das Fundament für die erfolgreiche Arbeit mit der Cloud gelegt. Die Cloud-Vision beschreibt, was langfristig mit der Cloud erreicht werden soll, wie die Cloud zur Weiterentwicklung des Unternehmens beitragen kann und welche Technologien benötigt werden. Basierend auf der Vision wird die Cloud-Strategie definiert. Diese enthält konkrete Ziele und sollte immer an der allgemeinen Unternehmensstrategie sowie der bestehenden IT-Strategie orientiert sein.

Die zweite Phase der „Cloud Transformation Roadmap“ umfasst die Planung und das Design der Cloud. Hier wird evaluiert, wie funktionierende Cloud-Prozesse und Technologien aussehen müssen und umgesetzt werden können. Die Durchführung von Workshops und Interviews mit internen Experten aus unterschiedlichen Bereichen trägt dazu bei, die benötigten Prozesse und Technologien für die Arbeit mit der Cloud zu definieren. Themen wie Security, Netzwerk, Anbieterwahl, IAM (Identity and Access Management) und Cloud Governance werden hier diskutiert. Um das Cloud Design festzulegen, empfehlen wir die Erstellung eines sogenannten Cloud Blueprints als Vorlage bzw. Zielbild für die Cloud. Hier wird das Cloud Design hinsichtlich der Prozesse (diese werden später im Cloud Governance Framework noch spezifiziert) und der Technologie beschrieben.

Es ist empfehlenswert, schon zu Beginn der Cloud-Einführung ein Proof of Concept (PoC) zu bestimmen, anhand welchem direkt getestet wird, ob die geplanten Prozesse, Richtlinien etc. auch tatsächlich umsetzbar sind.

Weiterhin sollte bei der Cloud-Planung die Ressourcenplanung berücksichtigt werden: Wie viele MitarbeiterInnen und wie viel Arbeitszeit werden benötigt? Welche zusätzlichen Kosten fallen an? Eine gute Ressourcenplanung und Aufwandsschätzung reduzieren die Gefahr für spätere Engpässe. Bereits in dieser Phase sollten die MitarbeiterInnen mit an Board geholt und entsprechend gebrieft und vorbereitet werden.

Bei der dritten Phase der „Cloud Transformation Roadmap“ stehen die Verantwortlichkeiten und Prozesse im Mittelpunkt. Hier gilt es, ein ganzheitliches Konzept auszuarbeiten, welches alle Rahmenbedingungen und die Regelung von Verantwortlichkeiten festlegt. Sämtliche Prozesse rund um die Themen Sicherheit, Betriebsmodell und das Management der Cloud werden klar definiert. Der Schwerpunkt der dritten Phase und ein essenzieller Bestandteil der kompletten Cloud Transformation ist die Erarbeitung eines Cloud Governance Frameworks, welches als Grundlage für die technische Umsetzung bzw. die Implementierung aller Prozesse, Mechanismen und Security-relevanten Themen dient (siehe Abschn. 3.2).

Die vierte Phase der „Cloud Transformation Roadmap“ dreht sich um die Festigung und die Expansion der Cloud. Die kontinuierliche Cloud-Optimierung steht hier im Vordergrund. Inhalte dieser Phase sind die laufende Unterstützung der Fachbereiche, Schulungen, das Thema Change Management und die stetige Weiterentwicklung des Cloud Governance Frameworks. Die Cloud Transformation ist also nie hundertprozentig abgeschlossen, denn es wird immer wieder Neuerungen und Anpassungsbedarf der Cloud geben.

Da sich die Anforderungen im Cloud-Bereich schnell ändern können, ist bei der Cloud Transformation ein agiles Projektmanagement zu empfehlen.

### Cloud Governance Framework

Das Cloud Governance Framework ist ein zentrales Element der Cloud Transformation. Es dient als Instrument für die Steuerung der Bereitstellung, Kontrolle, Verwaltung und den Betrieb der Cloud. Hier werden alle Rahmenbedingungen für den Cloud-Betrieb festgehalten (Strategie, Management Prozesse etc.), aber auch die Anforderungen an die Cloud Security definiert, Prozesse und eingesetzte Technologien festgehalten und Richtlinien für die Cloud-Nutzung definiert. Das Cloud Governance Framework dient als eine Art Guideline und unterstützt dabei, die Cloud zielgerichtet und effizient einzusetzen. Ohne das Cloud Governance Framework entsteht schnell ein unkontrollierbarer „Cloud-Dschungel“ aus Subscriptions, Nutzer- & Rechte-Vergaben und unterschiedlichen Security-Standards. Sämtliche Beschlüsse und Prozesse für die Arbeit mit der Cloud werden im Governance Framework festgehalten und in klare Regelungen und Verantwortlichkeiten übersetzt.

#### Richtlinien & Regeln für die Cloud

**Abb. 3:**
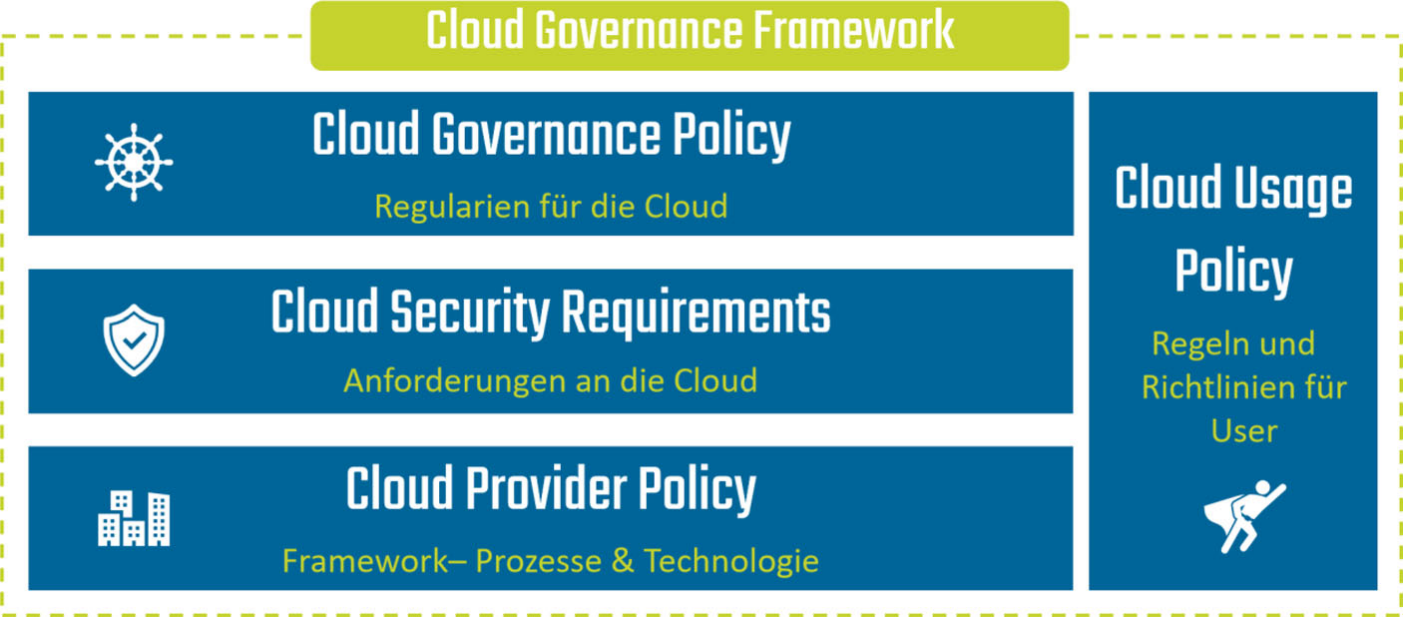
Inhalte des Cloud Governance Frameworks (eigene Darstellung)

Das Cloud Governance Framework lässt sich in vier Hauptbereiche unterteilen (Abb. 3).

- **Cloud Governance Policy:** Sammlung der Regularien für die Cloud (Strategie, Organisation, Account Management, Monitoring, Reporting etc.)
- **Cloud Security Requirements:** Anforderungen an die Sicherheit der Cloud (Standorte, Betrieb, Vertragliches, Datenschutz etc.)
- **Cloud Provider Policy:** Konkrete Ausgestaltung eines Provider-Frameworks für Prozesse, Technologien und Konfiguration einer spezifischen Cloud-Plattform
- **Cloud Usage Policy:** Regeln und Richtlinien für User zur konformen Cloud-Nutzung (Datenklassifizierung, rechtliche Voraussetzungen, Verantwortlichkeiten etc.), Definition wer wann was macht in Form einer RACI-Matrix^4^

#### Die Sicherheit der Cloud als zentrale Herausforderung

Die Themen Datenschutz und Datensicherheit spielen auch bei der Cloud eine wichtige Rolle und gelten bei der Cloud Transformation als zentrale Herausforderung für Unternehmen. Den Sicherheitsanforderungen an die Cloud sollte demnach sowohl bei der Cloud-Planung als auch bei der späteren Nutzung große Aufmerksamkeit geschenkt werden. Ein wichtiger Punkt hierbei ist die Vertragsgestaltung mit dem Cloud-Provider. Die Anforderungen an einen Anbieter sowie den Vertrag sollten vom Unternehmen definiert werden. Bei der Definition der Auswahlkriterien sowie der Vertragsgestaltung sollte sichergestellt werden, dass ausreichende Sicherheits- und Funktionsreserven für zukünftige Anforderungen an die Cloud gewährleistet sind.

Die *Internetanbindung* spielt eine entscheidende Rolle bei der Verfügbarkeit von Cloud-Diensten. Neben einer ausreichenden Verfügbarkeit über SLA (Service Level Agreement) ist zu beachten, dass auch redundante Internetleitungen ausfallen können. Eine Back-Up Anbindung bei einem alternativen Internet-Anbieter mit reduzierter Bandbreite sollte für die wichtigsten Standorte umgesetzt werden. Durch die aufgrund von Covid-19 weit verbreitete Homeoffice Arbeit bedeutet ein Ausfall der Internetverbindung auch kein Zugriff auf benötigte Systeme und keine Möglichkeit zur (Krisen‑)Kommunikation und Kollaboration.

Cloud-Dienste bringen neue Risiken mit sich, die im herkömmlichen IT-Betrieb meist nur eine kleine oder gar keine Rolle gespielt haben. So sind sie zum Beispiel Ziele für jede Art von *Cyber-Attacken oder -Kriminalität*. Das Sicherheitsniveau auf der Ebene der Redundanz und der physischen Sicherheit kann zwar viel höher sein als in den eigenen Rechenzentren, gleichzeitig ist aber die Wertdichte deutlich höher und damit auch die Wahrscheinlichkeit und Intensität eines potentiellen Angriffs.

Durch die Nutzung von Cloud-Diensten besteht immer eine gewisse Gefahr, dass der entsprechende Dienstleister seine Vorteilsposition, die sich aus den hohen Investitionen seiner Kunden ergibt, zum *wirtschaftlichen Missbrauch *ausnutzt. Er könnte zum Beispiel seine Margen durch massive Preiserhöhungen, Leistungseinschränkungen oder Qualitätsminderungen erhöhen oder Änderungen der Konditionen festsetzen, die für den Cloud-Nutzer unvorteilhaft sind.

Als weiteres Risiko kann eine potentielle *Übernahme des Dienstleisters* durch Dritte gesehen werden. Dies könnte zu grundlegenden Änderungen in der Ausrichtung und Handhabung des Dienstes führen, die nicht mit den eigenen Anforderungen vereinbar sind. Auch könnten Geschäftsmodelle der Datensekundärnutzung entstehen oder es könnte zur Einstellung des Dienstes kommen. Falls der neue Eigentümer des Cloud-Diensts einer anderen Gerichtsbarkeit unterliegt als der ursprüngliche Eigentümer, könnte die Durchsetzung zentraler Vertragsklauseln gefährdet oder unmöglich sein.

Um die Sicherheitsrisiken zu vermeiden oder leichter handzuhaben, sollten besondere Anforderungen an die Informations- und IT-Sicherheit sowie den Datenschutz für die Auswahl der Cloud-Dienste gestellt werden. Diese werden in den Cloud Security Requirements im Cloud Governance Framework festgehalten und betreffen diese Themen:

- Authentifizierung und Identitäten, Rollenmodelle
- Verbindungen, Netzwerk und Segmentierung
- Datenklassifikation und Datenschutz
- Backup und Recovery
- Logging und Auditing

Wie bei den Herausforderungen der Cloud Transformation in Abschn. 2.2 bereits erwähnt, gilt beim Thema Datensicherheit und Datenschutz die geteilte Verantwortung (siehe hierzu AWS o.J.; Microsoft 2021; Google 2019). Sowohl der Cloud Provider als auch der Cloud-Kunde sind hier für einen Teil der Sicherheitsaufgaben zuständig. Hat ein Unternehmen ein lokales Rechenzentrum, ist es selbst für alle Sicherheitsbelange zuständig. Bei einem Wechsel in die Cloud wird je nach Service-Modell ein unterschiedlich großer Anteil dieser Aufgaben vom Cloud-Anbieter übernommen. Die Daten und Identitäten gehören immer dem Cloud-Nutzer. Daher ist dieser auch für deren Sicherheit sowie für den Schutz der lokalen Ressourcen und der selbst gesteuerten Cloud-Komponenten zuständig.

Bei einer Nutzung von Cloud-Diensten sollte außerdem für den Ernstfall eine belastbare *Exit-Strategie *definiert werden.

### Erfolgsfaktor Change- & Adoption-Management

Wie bereits beschrieben, bringt die Einführung der Cloud grundlegende Veränderungen im Unternehmen mit sich. Solche Neuerungen erfordern immer ein professionelles und umfangreiches Cloud- und Adoption-Management (Kavis 2020, S. 158). Durch veränderte Prozesse und den Einsatz neuer Technologien müssen alle Mitarbeitenden umdenken, insbesondere auch die IT-Abteilung. Ein Beispiel: Hat man in der traditionellen On-Premise IT präventiv verschiedenste Maßnahmen getroffen, um den Ausfall einer Komponente zu verhindern, muss in der Cloud jederzeit mit dem Ausfall einer Komponente gerechnet und entsprechende Vorbereitungen getroffen werden.

Durch die Veränderungen der Arbeitsweise, welche durch Covid-19 gewissermaßen erzwungen wurden, mussten Mitarbeitende auch ihren Arbeitsort verlegen und teilweise neue Tools einsetzen. Unternehmen mussten dies entsprechend so gut es geht ermöglichen. Für Cloud Transformation sollte auf dieser Erfahrung aufgebaut werden. Hier muss nun nicht mehr bei Null angefangen werden, denn der Change hat schon begonnen.

Für eine effiziente und zielorientierte Nutzung der Cloud ist es wichtig, dass alle Nutzer die neuen Möglichkeiten und Strukturen der Cloud-Lösung kennen und wissen, wie sie die Cloud in ihrem Bereich richtig einsetzen. Mitarbeitende sollten daher frühzeitig in das Projekt miteinbezogen werden – zum einen durch die Information über die geplanten Neuerungen und den Projektverlauf, zum anderen durch ein aktives Einbinden unterschiedlicher Unternehmensbereiche. Damit werden die Bedürfnisse der Mitarbeitenden berücksichtigt, sie werden auf die Cloud-Einführung vorbereitet und können sich auf die Veränderung einstellen. Die Kommunikation von bereichsspezifischen Prozessbeispielen und Vorteilen kann das Commitment bestimmter Personen erhöhen. Allgemeine und auf bestimmte Anwendungen spezialisierte Schulungen sollten rechtzeitig und fortlaufend angeboten werden. Durch diese Maßnahmen wird die Akzeptanz der Mitarbeitenden für Veränderungen durch die Cloud erhöht und eine positive Einstellung gegenüber neuer Cloud-Lösungen gefördert. Ebenfalls ist es wichtig, den Betriebsrat frühzeitig in das Cloud-Projekt zu involvieren und mögliche Interessen des Betriebsrats als Mitarbeitenden-Vertretung zu berücksichtigen.

Ein frühzeitiger Aufbau von neuen Kompetenzen zählt ebenfalls zu einem erfolgreichen Change Management. Neues Knowhow muss aufgebaut und neue Rollen müssen geschaffen werden, auch in der IT-Abteilung. Durch die im Zuge der Corona-Pandemie entstandenen Möglichkeiten der Remote-Arbeit können Unternehmen offene Stellen eventuell auch ortsunabhängig rekrutieren. Durch die Ausweitung des Einzugsgebiets erhöht sich die Chance, geeignete Fachkräfte zu finden.

Ein erfolgreiches Cloud- und Adoption-Management bewirkt eine höhere Motivation und Bereitschaft aller Beteiligten, sich mit der Veränderung auseinanderzusetzen und diese als Chance zu sehen. Die damit einhergehende höhere Leistungsbereitschaft wirkt sich letztendlich positiv auf die Produktivität des Unternehmens aus. Das Change Management betreffend Cloud kann allerdings nicht als zeitlich begrenzte Aufgabe gesehen werden kann – die Cloud bringt immer wieder Neuerungen mit sich, die entsprechend kommuniziert und integriert werden müssen.

### Cloud Competence Center als Center of Excellence

Nicht nur die Umsetzung der einzelnen Use Cases, z. B. in Form von VMs (Virtuellen Maschinen), SaaS Applikationen oder einzelne Functions (FaaS), sondern auch die Cloud-Plattform an sich muss gemanagt werden. Themen wie Fragen zur Subcription-Struktur, interne Abrechnung (Billing), Cloud-Prozesse, Architektur oder zur Security unterliegen einer ständigen Veränderung und müssen regelmäßig für das Unternehmen validiert werden. Die Einrichtung einer zentralen Kontrollinstanz für diese Themen, welche entsprechende Fragen beantwortet, Best Practices definiert und die Umsetzung kontrolliert, ist daher sehr sinnvoll und ratsam. Diese Kontrollinstanz wird als Cloud Competence Center (CCC) bezeichnet und dient als zentrale Anlaufstelle für sämtliche Cloud-Angelegenheiten.

Das CCC fungiert als Cloud-Expertenteam und unterstützt mit seinen Fähigkeiten sowohl die einzelnen Fachbereiche als auch die IT-Abteilung. Bei der Cloud Transformation hilft es bei der Definition der neuen Rollen, z. B. des Cloud-Architekten oder des Cloud-Beraters. Es legt fest, was die neuen Verantwortlichkeiten und Aufgaben sind, aber auch wie die jeweiligen Personen zum neuen Knowhow kommen. Insbesondere für kleinere Unternehmen relevant ist, dass es sich beim CCC auch um ein virtuell aus verschiedenen Rollen zusammengesetztes Gremium handeln kann, das über verschiedene Standorte oder Abteilungen hinweg zusammengesetzt ist und nicht unbedingt aus Vollzeitstellen bestehen muss. Dass CCC muss keine extra Abteilung oder ein besonders großes Team sein. Wichtig ist die kompetente Abdeckung der Aufgaben und Funktionen des CCC.

Das CCC sollte als Center of Excellence gesehen werden. Seine Tätigkeiten zielen darauf ab, einen wesentlichen Beitrag zu einem Kulturwandel im Unternehmen beizutragen. Das Business bzw. die Fachbereiche sollen bestmöglich unterstützt und nicht gebremst werden. Anstatt dass die IT wie bisher als eine Art Ampel funktioniert, die auf Kontrolle und zentrale Verantwortung ausgelegt ist, liegt der Fokus neu auf der Freiheit und delegierter Verantwortung. Letzteres entspricht eher einem Kreisverkehr, in den jeder eigenverantwortlich rein- und rausfahren kann.

## Empfehlungen für eine erfolgreiche Cloud Transformation

Um die Cloud Transformation auch in Zeiten von Corona erfolgreich voranzutreiben und durchzuführen, kommt es auf ein strukturiertes, zielorientiertes Projektmanagement sowie ein ganzheitliches Change Management an. Hierfür lassen sich folgende fünf Empfehlungen ableiten, sie sich in der Praxis bereits mehrfach bewährt haben:

- **Bestimmung eines dedizierten Projektleiters: **Der Cloud-Projektleiter übernimmt die Projektsteuerung und behält den Überblick sowie den Blick von außen auf die jeweiligen Fachbereiche. Er treibt das Thema Cloud kontinuierlich voran.
- **Abholung des Managements: **Das Management trifft strategische und wegweisende Entscheidungen für das Unternehmen – die Entscheidung bzgl. Cloud zählt hier dazu. Deshalb ist der frühzeitige Einbezug des Managements und dessen Aufklärung über das Thema relevant für die Genehmigung für den Cloud-Einstieg sowie den Projekterfolg.
- **Schaffen von Transparenz: **Eine transparente und laufende Kommunikation über das Projekt ist zentral für ein erfolgreiches Change Management. Hier kann zum Beispiel die Einführung eines Cloud-Portals helfen – eine Art Intranet für Cloud-Themen.
- **Aufbau eines Cloud-Teams (CCC): **Das CCC beschäftigt sich intensiv mit grundlegenden Cloud-Themen und dient als internes Beraterteam (siehe Abschn. 3.4).
- **Umsetzung von Proof-of-Concepts und Pilotprojekten: **Bereits während der Erstellung des Cloud Governance Frameworks (siehe Abschn. 3.2) sollte mit der parallelen Umsetzung von Proof-of-Concepts bzw. von Pilotprojekten gestartet werden. So werden frühzeitig die Praxistauglichkeit des Frameworks auf die Probe gestellt und bereits erste Ergebnisse sichtbar. Auf diese Weise kann die Akzeptanz für das Thema Cloud zusätzlich gefördert werden.

## Fazit und Ausblick: Was bringt die Zukunft?

Zusammenfassend lässt sich festhalten, dass die Cloud Transformation durch Covid-19 nicht komplett verändert wurde – die Pandemie ist jedoch eine weitere Motivation und für einige Unternehmen auch ein Zwang, sich *jetzt* und nicht erst in ein paar Wochen oder Jahren mit dem Thema Cloud auseinanderzusetzen.

Es wird zukünftig immer mehr Cloud-Services geben, ihr Einsatz wird weiter zunehmen. Daher gewinnen die Definition klarer Strukturen, Prozesse und Konzepte für die Cloud Transformation, sowie die eindeutige Regelung der Verantwortlichkeiten, zunehmend an Bedeutung. Viele Unternehmen führen selbst neue, cloudbasierte Geschäftsmodelle ein, einige Innovationen sind nur mit Hilfe von Cloud-Technologien umsetzbar.

Die Auswahl der richtigen Services und des richtigen Providers wird in Zukunft darüber entscheiden, wie effizient Services für das Unternehmen eingesetzt werden können.

Eine erfolgreiche Cloud Transformation ist aufwendig, sie braucht viel Zeit und Ressourcen. Wenn sie aber durchdacht durchgeführt wird, d. h. mit Planung, strukturierten Prozessen und einem ganzheitlichen Konzept, dann wird sie Unternehmen langfristig und nachhaltig unterstützen.

## Footnotes

1 Beim *Greenfield Approach* wird eine neue Anwendung oder Technologie entwickelt, ohne dabei die Entwicklungen aus der Vergangenheit zu beachten (Wade 2018).

2 Unter Cloud Sprawl versteht man allgemein das Durcheinander existierender Ressourcen, Accounts und Gruppen.

3 Ein lokaler Internet-Breakout ist ein Zugangspunkt zum Internet, der sich möglichst nah am Benutzer befindet. Lokale Breakouts ermöglichen Unternehmen, internetgebundenen Datenverkehr von lokalen Zweigstellen und Außenstellen auszulagern und ihn über einen lokalen Internet Service Provider (ISP) direkt ins Internet zu leiten (Zscaler o.J.).

4 RACI steht für Responsible (engl. verantwortlich), Accountable (engl. rechenschaftspflichtig), Consulted (engl. konsultiert) und Informed (engl. informiert). Eine RACI Matrix bildet Rollen und Verantwortlichkeiten innerhalb eines Projekts ab. Sie definiert, wer für welches Aufgabengebiet verantwortlich ist und welche Personen wann zu Rate gezogen und informiert werden (Stein 2009, S. 87).
